# Registered nurses’ knowledge, attitude and practice regarding their scope of practice in Botswana

**DOI:** 10.4102/hsag.v25i0.1415

**Published:** 2020-10-20

**Authors:** Maria M. Feringa, Hester C. de Swardt, Yolanda Havenga

**Affiliations:** 1 Adelaide Tambo School of Nursing Science, Faculty of Science, Tshwane University of Technology, Pretoria, South Africa

**Keywords:** Attitude, Botswana, Guidance, Knowledge, Registered nurses, Scope of practice

## Abstract

**Background:**

The articulation of the scope of practice in nursing is important to provide boundaries for registered nurses in which to practice. Registered nurses in Botswana have frequently experienced challenges and raised concerns with their scope of practice. Research related to registered nurses’ knowledge, attitudes and practice regarding their scope of practice appears to be limited in the African context, particularly in Botswana.

**Aim:**

The aim of this study was to develop guidelines for professional nurses to explore and describe registered nurses’ knowledge, attitude and practice regarding their scope of practice in Botswana.

**Methods:**

A convergent parallel mixed-methods design was employed using a three-tier sampling approach to ensure a representative sample of various settings, health facilities and nurses. For the purpose of this article, the data from the qualitative component are reported. Thirty registered nurses, working in the public health sector in Botswana, participated in semi-structured interviews. Data were analysed using thematic content analysis.

**Findings:**

Data analysis revealed that registered nurses’ scope of their knowledge was lacking. Registered nurses’ attitudes were reflected in the adaptation process to expanded practice, as demonstrated through emotive aspects, adjustments to practice beyond scope and the learning of new skills considered beyond scope. Participants reported implementing many skills deemed beyond their scope, whilst their motive to do so included their experience of a lack of control over practice, lack of resources or they were doing so out of consideration for the patient. Guidance in terms of their scope was found to be inadequate.

**Conclusion:**

As in other resource-limited countries in Africa, registered nurses in Botswana experience challenges with their scope of practice. Inadequate boundaries may result in compromised nursing care and may have detrimental consequences for both the patient and the nurse.

## Introduction

Nurses’ scope of practice outlines the boundaries in which registered nurses (RNs) are expected to practice (ICN 2014). Boundaries are determined by those interventions that RNs (also referred to as ‘nurses’ in this article) are legally recognised, educated and competent to implement (Altranais 2000). A clear articulation of the scope of practice is important to the nursing profession to ensure safe and quality practice, endorse the identity of the nursing profession and promote the most effective use of resources (ICN 2014). A scope of practice, furthermore, allows RNs to practice efficiently to their fullest extent and capabilities (ICN 2014), whilst protecting the patient and the nurse.

Internationally, the scope of practice of RNs has evolved and expanded considerably during the past decades; this has, however, not always gone hand-in-hand with legislative support, such as regulations or protective policies (ICN 2010; McCarthy et al. 2013b; Spies 2016). When new health programmes are introduced in Africa, these have frequently been allocated to RNs. Tasks are also expected to continue to shift to RNs in the foreseeable future, for instance, in the context of non-communicable diseases (Some et al. 2016). Registered nurses’ capability to successfully expand their scope is amply exemplified by the HIV epidemic in Africa (Mwijarubi 2015).

## Background

Nurses in Botswana comprise the majority of healthcare workers and are considered, like elsewhere in Africa, (Ng’ang’a & Woods-Byrne 2012) to be the backbone of providing the bulk of health services. Registered nurses often perceive themselves to be working outside their scope of practice, not least because of the persistent difficulty in demarcating their responsibilities within a legal and professional framework. The frustration of RNs with the lack of clarity concerning their scope of practice is further shaped by the historical context from which nursing in Botswana evolved (Miles et al. 2007; Selelo-Kupe 1993). Over the years, shortages of allied health professionals and medical practitioners have resulted in expansive task shifting towards RNs (Miles, Seitio & Mcgilvray 2006), which remains relevant today. Nurses are often responsible for duties and interventions they consider to be beyond their scope or regard as ‘non-nursing’ duties; this is a finding congruent with other parts of sub-Saharan Africa (East et al. 2014; Ng’ang’a & Woods-Byrne 2012). Consensus between RNs and the employer about which interventions are considered to fall within the scope of practice appears wanting. The need for scope of practice clarification for RNs has thus been an ongoing concern, especially amongst RNs in the public service and their employers. Registered nurses, at times, feel overwhelmed, not recognised and experience stress because of potential risks, which may contribute to their level of motivation and job satisfaction (Spies 2016; Spies et al. 2016).

In many sub-Saharan countries, RNs have historically practised beyond their scope of practice in order to meet the health needs of populations, usually because of challenges with adequate human resources in health (APPG 2016; Mijovic 2016; Spies 2016), currently referred to as ‘task shifting’ (Baine & Kasangaki 2014; Terry et al. 2012). In Botswana, similarly, a pattern of RNs making medical decisions and prescribing medications (Boonstra et al. 2002, 2003; Boonstra, Lindbaek & Ngome 2005) because of limited resources was established over time, a trend which continues, especially in rural and remote areas (Miles et al. 2006).

Nurses, as a professional cadre, are generalists who can be deployed in many settings and are more economically affordable compared with several other and often more expensive professionals employed in the health sector (Grosso et al. 2019). Tasks are therefore expected to continue to shift to RNs in the foreseeable future to meet the health needs of populations; hence, the evolvement of the scope of practice appears inevitable. The number of studies that have investigated the actual scope of practice of RNs, specifically in African countries, remains limited (Benton et al. 2017; Oelke et al. 2008). Information is thus lacking in both role and task analysis for RNs and midwives (Seboni et al. 2013).

Knowledge about the scope of practice of RNs and the degree to which RNs are ‘correct’ in terms of interpreting their scope of practice is scarcely documented. The RNs’ inability to clearly articulate their scope of practice is also evident in literature (Aroke 2014; Lilibridge, Axford & Rowley 2000). Many RNs appear to understand the scope of practice to reflect what nurses actually do whilst performing day-to-day tasks (Besner et al. 2005; White et al. 2008), but they recognise that education, experience and competence influence the enactment of their scope.

Nurses’ attitudes towards their scope of practice appear mostly positive in literature, yet articles frequently relate to the introduction of specific new aspects or roles, expansion or extension of practice, rather than scope of practice in general (Ganz, Toren & Fadlon 2016; Senior 2008), such as, for instance, prescribing (Bhanbro et al. 2011; Buchan & Calman 2009; Lockwood & Fealy 2008). Articles from the African continent, addressing RNs’ attitudes to scope of practice, reflect both positive and negative perceptions. For instance, Spies (2016) identified conflicting roles and expectations amongst nurses in East Africa that were inconsistent with their scope of practice.

Nurses’ practice often appears to be below (under-utilisation) or beyond (above) scope (D’Amour et al. 2012; Nathenson, Schafer & Anderson 2007; Schluter et al. 2011). Moreover, the overlapping roles of health workers (within nursing and across disciplines) appear prevalent. Grosso et al. (2019) concluded that nurses, in their daily practice, occupy various non-nursing roles below, above and horizontal to their competencies. They found (Grosso et al. 2019) that RNs function both in and outside other healthcare professional spaces and that the nurse’s role appears to be a contingent one. In Africa, nurses are frequently forced or feel obliged to practice beyond their educational preparation and expand their practice to meet the health needs of the communities (Msuya et al. 2017). Similarly, in Botswana, RNs have experienced pressure from councils (municipalities) to work beyond their scope of practice because of inadequate numbers of other healthcare professionals (Linn, Wilson & Fako 2016).

### Problem statement

The absence of guidelines and frameworks in Botswana to guide and support RNs in decision-making when concerns or dilemmas regarding their scope of practice occur, may result in unsafe practices, risk-taking or potential exploitation, consequently compromising patient care (Boonstra et al. 2002, 2003; Boonstra, Lindbaek & Ngome 2005; Grosso et al. 2019; Lubbe & Roets 2014; Zachariah et al. 2009). In Botswana, RNs often perceive themselves to be working beyond their scope of practice, and there is little consensus between them and their employer regarding which interventions are considered within their scope. No formal study has been conducted in Botswana to elicit the knowledge, attitudes and practices of RNs in the public sector regarding their scope of practice to inform the development of guidelines to guide and support nursing practice.

### Aim and objective

The overall aim of the study was to develop guidelines to inform and direct the implementation of RNs’ scope of practice in Botswana, in order to promote improved health outcomes for nurses and patients. The specific objective reported in this article is to explore and describe RNs’ knowledge, attitude and actual practice regarding their scope of practice in the public health sector of Botswana.

## Methods

The study was exploratory and descriptive in nature. A convergent parallel mixed-methods research design (Creswell & Plano Clark 2018) was used. This design was adopted based on philosophical assumptions of the pragmatic paradigm (Creswell & Plano Clark 2018). The study took place in Botswana, amongst RNs in the public health sector, where the majority (approximately 90%) of the nurses are employed. In order to explore and describe the RNs’ knowledge, attitude and actual practice regarding their scope of practice in the public health sector in Botswana from an inductive and qualitative perspective, a three-tier sampling approach was designed, comprising a combination of quota-, stratified-, purposive- and convenience- sampling (Polit & Beck 2021). The quota sample resulted in one referral hospital (out of three) and 10 health districts (out of 27) throughout the country. A stratified random sample resulted in the inclusion of 30 purposefully and conveniently selected RNs from referral, district and primary hospitals, clinics and health posts. A sample size of 30 was considered as constituting an acceptable number (Adler & Adler 2012; Dworkin 2012).

Inclusion criteria included practising RNs in selected settings and facilities, registered with the Nursing and Midwifery Council of Botswana (NMCB), providing direct patient care, willing and available to participate in the study. Although saturation (Gray, Grove & Sutherland 2017; Malterud, Siersma & Guassora 2016; Polit & Beck 2021) was achieved after 19 interviews, the researcher continued until 30 interviews were completed to include nurses from all districts and settings of the country. The three-tier sampling approach ensured that the sample reflected the geographical extent and complexity of the country as well as the type of settings (from urban to remote) and facilities (from referral hospitals to health posts) where nurses were employed. Participants varied in age from 21 to 55 years, had been working from 1 to 26 years and were employed in various types of clinics and hospitals in urban, rural and remote settings throughout Botswana.

All interviewees were provided with an information leaflet, after which verbal and verbatim consent was obtained. A pre-tested, semi-structured interview guide was used to collect data, including prompts addressing participants’ knowledge, attitude and practice. The main question posed to participants was: ‘I am trying to elicit information that addresses your scope of practice, as you practice it. Please tell me about **your** scope of practice’.

Data were gathered over a period of 1 year, from March 2016 to March 2017. The interviews were conducted on an individual basis by the researcher, in a separate room at the health facility, in English, and they were digitally recorded. Interviews lasted from 13 to 26 min. Thereafter, audio recordings were transcribed verbatim.

Thematic content analysis was used to analyse the data. This process consists of identifying patterns or themes within qualitative data (Maguire & Delahunt 2017), as well as categories and subcategories. In this study, themes were pre-identified as knowledge, attitude and practice, parallel to the quantitative aspect of the study. The thematic content analysis also facilitated in the identification of additional themes, categories and subcategories. Codes were allocated to quotations, emanating from the interviews, to discern between participants. For instance: M3-24, where M represents the district where the interview took place, the number 3 the third participant within that district and 24 refers to the number given to the transcript.

### Ethical considerations

Approval for the implementation of the study was obtained from the Tshwane University of Technology (Ref. #: FCRE 2015/06/026 [SCI]) in South Africa and the Ministry of Health and Wellness in Botswana (Ref. #: HPDME 13/18/1 X [78]). Further permission to conduct the study was obtained from Chief Medical Officers in Health Districts in Botswana, whilst consent was obtained from the participant RNs. Participants were informed that the interview could be stopped at any time or that they could withdraw from the study if they wished to do so. Interviews were coded and reported in an aggregated format to ensure anonymity and confidentiality.

### Trustworthiness

Criteria to ensure trustworthiness were applied, inter alia, by spending sufficient time in the field, by including RNs from various settings and facilities in the country, and through member checking (Lincoln & Guba 1985 in Polit & Beck 2021), whereby two interviewed RNs re-read their transcripts. In addition, to determine inter-coding reliability (Polit & Beck 2021), 10 transcribed interviews were co-coded by an independent coder. Codes from the co-coder and the researcher appeared to be similar. A dependability and confirmabilty audit trail was kept and dense descriptions were provided to enhance transferability (Polit & Beck 2021).

## Findings

The question and prompts raised a wealth of information for analysis. The three pre-determined categories related to knowledge, attitude and practice were complemented by an additional category, which was labelled ‘Guidelines regarding scope of practice’. The four categories resulted in 12 subcategories, as depicted in Table 1. The subcategories are presented in an integrated manner to illustrate their interrelatedness.

**TABLE 1:** Overview of categories and subcategories.

Category	Subcategories
Knowledge deficit amongst RNs of their scope of practice	Limited understanding of scope of practice
	Limited knowledge of determinants of scope of practice
	Indecisiveness and insecurity regarding scope of practice
Attitude related to the adaptation process of the scope of practice to an expanded nursing practice	Emotive aspects
	Adjusting to practice beyond scope
	Learning skills beyond one’s scope
Registered nurses’ current practice related to motive and context	Interventions beyond scope of practice
	Motive to go beyond scope of practice Lack of control and resources Regard for the patient
	Difference between the hospital and clinic setting
Guidance regarding scope of practice in terms of boundaries	Unhappy with job descriptions
	Inadequate parameters
	Dealing with dilemmas

### Category 1: Knowledge deficit amongst registered nurses of their scope of practice

From the data, it emerged that a knowledge deficit existed amongst RNs regarding their scope of practice. This knowledge deficit referred to the fact that participants did not comprehend the essence of the scope of practice, appeared unfamiliar with the term or was rather cautious in their attempts to ascribe a meaning to the scope of practice. The knowledge deficit was demonstrated through the following subcategories: (1) limited understanding of scope of practice, (2) limited knowledge of determinants of scope of practice and (3) indecisiveness and insecurity regarding the scope of practice.

Limited understanding was reflected in the sense that the term ‘scope of practice’ was repeatedly addressed as ‘job descriptions’, ‘standard operation procedures’, ‘required standards of practice’ or ‘following the rules and regulations’. The job description was mentioned by many participants as determining one’s scope: ‘The job-description tells the day-to-day functioning of me as a nurse … But anyway, it’s not very clear as to what I should do’ (C1-5). Participants were unsure whether RNs in Botswana have a scope of practice as one participant explained ‘… I don’t think we have; we are doing everything’ (D1-15). The limited knowledge of scope of practice was further illustrated by the lack of and, at times, controversial information brought forward by participants of what determined someone’s scope of practice. Participants shared ‘policies and guidelines’, ‘the pledge, standards and certificate, … and sometimes there will be that savingram [official administrative communication]’ (D3-17). The Nursing and Midwifery Act (Botswana 1995) and its subsequent published rules and regulations (Botswana 2011) were rarely mentioned. Participants’ limited knowledge was exacerbated by their indecisiveness regarding the scope of practice and being insecure about whether they worked within or outside their scope: ‘… we are just doing, we think it’s nursing, so we are not quite sure about our scope of practice’ (B3-13). Not being confident about what to do and what not to do regularly emerged during interviews: ‘We don’t even know if we are protected or not’ (K1-30) or ‘… but it’s like we are just being mixed up now with other people’s roles or duties’ (F1-27).

### Category 2: Attitude related to the adaptation process of the scope of practice to an expanded nursing practice

Registered nurses experience difficulties whilst adapting to practice, which influence their attitudes towards practice. This is expressed through emotive aspects (emotions), the process of adapting to practice beyond scope and the learning of new skills. Moreover, participants agreed that adaptation occurred, albeit not always in a constructive or satisfactory manner. Three subcategories emerged as relating to the adaptation process, which were labelled (1) emotive aspects, (2) adjusting to practice beyond scope and (3) learning skills beyond one’s scope.

Emotive aspects comprised the feelings participants said they experienced when dealing with scope of practice issues. Emotive states were expressed by all participants as occurring regularly. Many diverse feelings emerged during the interviews, ranging from ‘insecurity’, ‘fear’, ‘unsafe’, ‘unprotected’, ‘coercion’, ‘risky’, ‘scared’, ‘ambivalent’, ‘unclear’, ‘conflicts/confusion’, ‘uncomfortable’, ‘un-informed’, ‘de-moralising and demotivation’, ‘worried’, ‘unappreciated’, ‘ignorance’ and ‘disgruntling, anxious’. Feelings were not necessarily directly expressed but sometimes implied in responses, such as:

‘[*A*]s a nurse you know you have the expanded role, it’s not your role but you have to do it … a patient didn’t get help and yet you are there, you can be called into custody … in this given situation you have to.’ (H2-25)

However, positive feelings were shared as well, including: ‘You feel proud of yourself after saving a life …’ (D2-16), and ‘… as time goes on you get used to … and you get comfortable doing it …’ (D3-17) while ‘… as a nurse you have that human heart’ (H1-24).

Although a gap was identified between education and practice, ‘Work is different when coming out of school …’ (B3-13) and ‘… not prepared for clinic work’ (B2-12), nurses expressed that over time they adapted to what they experienced as working beyond their scope. Participants felt that they had little or no choice in whether they agreed or not, liked it, or were comfortable working beyond their perceived scope:

‘[*H*]ave to go with the system/no option (C3-9), … as time goes on, you get used to and you get comfortable doing it (D3-17) … you own up the thing that you know you are not supposed to own, just for the sake of the patient.’ (F1-27)

Participants shared that they adapted to the expanded RNs’ role by learning various interventions and skills whilst working in practice or through continuing professional development (CPD) activities, which had not been taught at school. Examples of how these skills were learned varied, and included, inter alia, from other staff, just through practice, on the job, in-house training from other nurses, through workshops (with or without having received a certificate) and through observation. In addition, some participants stated: ‘… the guidelines are there (B1-14), the treatment guideline for Botswana … used in primary health care by doctors and nurses’ (E3-22). Or, as one participant pointed out: ‘… but when you get in the field, what you doing now, you were not taught in school, so you have to learn, while you are working’ (B2-12).

Some discrepancy emerged between RNs regarding specific skills learned in school and in practice. Several participants stated: ‘…some skills we just learned on the field there, gained the experience (D2-16) … in school we were just doing preparation’ (D4-18). Other participants claimed to have learned the skills during nursing education and training, ‘Yes, we were taught at school … and then there was a practicum on that …’ (E2-21) and ‘… the new coming students, they are saying now they are told they should prick, but in our days …’ (E1-20).

### Category 3: Registered nurses’ current practice related to motive and context

Category 3 addressed RNs’ current practice and comprised of (1) interventions beyond scope of practice, (2) nurses’ motive to go beyond scope of practice, consisting of two subcategories – lack of control and resources and regard for the patient – and (3) differences between hospital and clinic settings. Most participants agreed that, at times, they worked beyond their scope of practice, assuming the doctor’s role, the role of a pharmacist or that of a lab technician:

‘[*T*]here are many, many instances whereby you find yourself doing what somebody else should be doing (C2-6), … consultation … I think it should be done by a Medical Officer … and yeah, we do prescribe medications (C3-9). You find that the nurse does a job for everyone … a porter, a lab technician, a social worker … you are everything (E2-21) … when it comes to supplies, maintenance, I don’t have knowledge about those items.’ (C1-5)

Participants also expressed taking on the role of midwife; ‘… we attend to maternal cases … … that is beyond our scope, then we are forced to deliver because we are alone …’ (C4-8).

Participants believed that working outside the scope of practice was more pronounced for RNs practising in clinics ‘… I used to work in a clinic, before I came here [hospital setting]; so that’s when I knew that we are going beyond our scope …’ (A2-4) and ‘… we do insert cannulas, we collect blood … we do that on a daily basis’ (A3-1). Whilst another participant deployed at a clinic stated:

‘I think some of them they do not fall in my scope cause … Mmmm, the pharmacy issues, I think they fall under somebody else but I do them. I do order drugs, I do take stock, and then even issuing of those drugs …’. (C1-5)

The motive to go beyond one’s scope reflects the various reasons indicated by participants to justify the step to go beyond their parameters. The reasons to do so appeared two-fold: (1) lack of control and (2) regard for their patients. Participants expressed that they had little power over refraining from stepping beyond their scope of practice. Motives to do so included a lack of resources (shortages) or to avoid trouble. Participants felt they had no option but to practice beyond their scope of practice; ‘…there is a shortage of staff or may I say doctors to even help us (H3-26) … we don’t have the BP [blood pressure] machines, we don’t have thermometers’ (E2-21). Nurses also felt coerced to practice outside their scope, as one explained ‘that’s how the system is’ (C3-9). Another motive for practising beyond their scope of practice was related to the last term in the job description, which reads ‘any other related duties’; interpreted by the participants as implying any and everything. They explained, ‘Any other duties means that you do everything’ (A2-4). Being compelled to do so was further expressed by a participant stating, ‘because we don’t have a choice’ (A5-7). Nurses may feel compelled to work beyond their scope of practice to avoid possible consequences, such as the ‘fear of losing [their] job’ (C2-6), or preventing legal action, as one participant explained: ‘So, I am supposed to be doing that cause if I don’t, there would be a case’ (B2-12). Shortages of allied professional staff were also keenly felt by RNs, further compelling them to work beyond their scope of practice: ‘… whereby now we have to do other people’s job … we are doing everybody’s thing’ (D3-17).

Taking the patient into consideration was a motive for RNs to go beyond their scope of practice. Many participants offered empathically and supportively inclined motives for acting beyond the scope of practice by putting the patient or client at the centre of care. The need to help the patient or out of respect and benefit for the patient, made these RNs go beyond their scope of practice:

‘At the end of the day it’s about a patient to be helped, so if you just leave and don’t do those things … a patient will suffer, so we are determined reaching our goal of helping the patient.’ (E1-20)

Another participant explained: ‘You end up just out of respect and also just trying to help cause you are there to help that patient, you end up giving …’ (A2-4).

Nursing practice was found to be considerably different in hospitals and clinics. Several participants stated that in a clinic, the emphasis was on ‘… consulting, prescribing, procedures, managing resources’, whereas in a hospital setting, care was primarily directed at bedside nursing. A participant, currently employed in a hospital setting, stated:

‘[*I*]f you are in a hospital you know, I can go up to this level because there will be doctors, everybody will be there, but when you get outside in the clinic, you will be there alone and you’ll be everything and that’s where you go even way beyond your scope because you don’t have anybody to help you with that.’ (D3-17)

Conversely, a participant currently working in a clinic setting volunteered:

‘Yes. It is very different because there are a lot of challenges … it was the doctor who saw the patients … prescribed … there I was just doing bedside nursing … but here it’s out-patients … I have to prescribe, you have to do a lot of things, engage with the community, do home visits, go out for calls … eeh, there is a big gap.’ (B3-13)

### Category 4: Guidance regarding scope of practice in terms of boundaries

Category 4 addressed guidance in terms of boundaries. Participants generally agreed that such guidance was insufficient: ‘If there is one [a guiding document] I haven’t seen it …’ (B2-12) and ‘… I am not sure, maybe there are, it’s just that I don’t know of them, yeah, so I am not sure’ (K2-29). The subcategories that emerged from the data included (1) participants being unhappy with job descriptions, (2) inadequate parameters and (3) dealing with dilemmas.

Participants indicated that they considered their job description as the major determinant of their scope of practice. However, they emphasised that the last portion of the job description, which states ‘Any Other Duty’, should be elaborated on, specified or removed. Participants explained: ‘Any Other Duties means that you do everything’ (A2-4) and ‘You never know what this “any other related duties” is’ (C3-9). Participants expressed that ‘any other duties’ were used as a pretext by the employer to justify going beyond one’s scope of practice and contributed to an underlying sense of insecurity regarding nurses’ scope of practice.

Current existing regulations were perceived as being inadequate to guide RNs in practice and were not protecting them sufficiently, which was explained as follows:

‘[*I*]t’s not enough to guide me (B2-12), … is not covering us (D4-18) … I think there is a little bit of it … though it does not cover some areas … the clinics (D1-15) … we should have a direction where we are in our service, we should know where we end (C2-6) … should be very clear in writing.’ (D2-6)

The notion of being unhappy with part of their job descriptions and having inadequate parameters contributed to the dilemmas nurses faced when trying to determine whether certain interventions fell within their scope, expressed as follows:

‘How we are being covered if something went wrong (K1-30), if it backfires you are wrong, if it prospers, they cherish you’ (H2-25) and ‘if a patient comes and you say no it is not within my scope of practice, … you can be in trouble, … you’ll have a dilemma.’ (C2-6)

Nurses expressed that they needed a sense of security in terms of where they were at, what was allowed or not allowed and to what extent they were covered.

## Discussion

The findings illustrate and emphasise the challenges nurses in Botswana experience with their scope of practice. Findings repeatedly indicated that RNs’ knowledge about the boundaries of their practice was inadequate. Nurses’ inability to articulate their scope of practice clearly, in terms of facts, understanding and application, has been acknowledged (Aroke 2014; White et al. 2008), whereas boundaries appear to be poorly understood (Birks et al. 2016; Oelke et al. 2008). An understanding of the scope of practice is essential for the provision of safe, effective and quality care (Bell 2005), the effective utilisation of the workforce (Besner et al. 2005) and the prevention of employment consequences (Brooke 2009).

Similar to the varied interpretations on the scope of practice in this study, differences amongst nurses in the interpretation of their scope were found by Brady et al. (2015). White et al. (2008) reported that nurses most often discussed the scope of practice by referring to the tasks they perform, rather than the roles they play in healthcare, thus supporting the findings in this study where RNs often viewed their job descriptions as similar to their scope of practice. Spies (2016) found that nurse leaders repeatedly referred to nurses’ scope of practice as routine nursing responsibilities, whereas Schluter et al. (2011) claimed that some participants referred to job descriptions as being scope of practice, which appears consistent with the findings of this study. Participants moreover failed to identify the Nursing and Midwifery Act (Botswana 1995) – and the rules and regulations that augment the Act (Botswana 2011) – as being part of what determines the scope of practice. Such oversight may have detrimental consequences in terms of, for instance, legal issues (Lockwood & Fealy 2008; McCarthy et al. 2013a; Mijovic 2016). A sense of indecisiveness and insecurity about their scope of practice further emerged from the study. Participants were unsure about which interventions fell within and outside their scope. This appears consistent with findings discussed by Byrne (2015) in an integrative review, who found discordance between actual and expected nursing practice in terms of non-nursing roles being performed by nurses.

Attitudes were reflected through a process of adaptation, but again, discordance was discovered in the process of doing so, as confirmed in literature (Spies 2016; Spies et al. 2016). Learning and adjusting were perceived not to be natural, but more often than not, participants felt compelled to go with the system, instead of considering their own professional opinion. However, there was some awareness and understanding of why RNs were adapting in that particular way. The emotive aspects that emerged from the interviews are illustrative of a challenging and not always satisfactory adaptation process. Sometimes emotional well-being (feeling comfortable) prevailed, but emotional discomfort predominantly seemed to have an upper hand (being uncomfortable or insecure). The findings are consistent with the emotive dissonance nurses may find themselves in, as addressed by Spies (2016). Conflicting emotional states are further confirmed by Iwu and Holzemer (2017), who stated that nurses’ job satisfaction increased because of task sharing in the context of HIV, whilst significant challenges simultaneously occurred because of the demanding nature of their new role. Emotions influence nurses’ adaptive process and, consequently, their performance. For instance, employees who experience mainly negative emotions during work may sooner experience burn-out or job dissatisfaction (Bakker & Oerlemans 2010).

Participants, however, felt that over time they adjusted to the system. Many skills considered to be beyond the scope of practice were learned on the job, not through a formal process, as is consistent with the findings by Spies (2016). As in Botswana, Spies (2016) found that most nurses were not fully prepared but instead relied on their basic education and observation of others whilst in their initial positions. According to Brady et al. (2015), developing and/or maintaining competence is of concern amongst nurses and midwives, especially in an environment of limited resources and where re-deployment is common. Both of these factors apply to the Botswana context (MOH 2010).

Attitude about one’s scope of practice may fluctuate. Negative attitudes could result in compromised nursing care, not only affecting the quality and safety of patient care, but also the motivation and job satisfaction of nurses themselves (Iwu & Holzemer 2017). Literature indicates that attitudes towards extended or expanded practice often appear positive (Lockwood & Fealy 2008) when going hand-in-hand with sufficient training, support and legal cover. However, conflicting roles and challenges have ensued (Iwu & Holzemer 2017; Spies 2016).

Ideally, nurses practice according to their optimum scope, that is, to the fullest extent of their education and competence not only to provide quality and safe patient care but also to enhance job satisfaction (ICN 2014; McCorkle et al. 2012; Powers 2013). In reality, however, RNs may practice below or beyond their scope (Nathenson et al. 2007; Upenieks et al. 2007; Schluter et al. 2011). Working beyond one’s scope of practice may result in malpractice and could demotivate nurses (Mathauer & Imhoff 2006) as they may feel challenged in their work performance (Iwu & Holzemer 2017) and even experience a sense of exploitation (Mijovic 2016). Conversely, working below one’s scope of practice may result in loss of skills. In this study, interventions beyond scope of practice were identified, and included, inter alia, taking on roles which normally would be the prerogative of other members of the multidisciplinary team and support staff. Nurses claimed not to be content with taking on these roles, feeling these belonged to other staff. Spies (2016) similarly found that nurses were expected to take on the work of others, which was considered a ‘heavily laden’ subtheme in her study. Nurses still perform many non-nursing duties and tasks, which could be done by less qualified staff (Bruyneel et al. 2013; Grosso et al. 2019). However, in Tanzania, Msuya et al. (2017) found that nurses (*n* = 166) at all levels of education, whether additionally trained or not, were prescribing (average 68%, whereas in rural clinics 94%) and performing minor surgical procedures (84%), well beyond their level of education.

The motive to go beyond one’s scope appeared two-fold; on the one hand, many negative elements emerged, and on the other, positive aspects surfaced. Spies et al. (2016) similarly found positive and negative aspects resulting from expanded scope of practice amongst nurses in Uganda. According to Spies et al. (2016), nurses’ perceptions to go beyond their scope of practice are complex and engulfed in ambiguity. This may result in discordance within and amongst nurses and their employer, leading to decreased job satisfaction. Lack of control over one’s practice also emerged as nurses felt they had no direct power over making professional judgements regarding their scope. They felt compelled to work in a system with which they are unhappy, causing them demotivation and job dissatisfaction. Spies (2016) found that nurses in East Africa repeatedly referred to a need to more clearly describe routine nursing responsibilities.

Lack of resources, particularly human resources, contributed to the nurses’ feelings of being ‘a jack of all trades’. In the absence of other qualified health staff, nurses were expected to take on the roles of these healthcare workers, including those of doctors, pharmacists and lab technicians, without having received appropriate training and support or financial compensation. These findings are confirmed by Spies (2016), while Mijovic (2016) adds that performing tasks beyond one’s scope may potentially compromise patient safety. However, some ambiguity between participants emerged as to what interventions belonged to scope of practice. Interventions that were regularly elaborated on and considered as being outside the scope of, for instance, consulting, prescribing, dispensing, cannulation and taking blood, were negated by some nurses, who stated that they did receive training to do so. Literature also describes task shifting or sharing, usually for economic reasons, as the reason to work beyond one’s scope (Spies 2016; Zachariah et al. 2009). Although task shifting, as such, did not directly emerge from any of the participants, indirectly nurses referred to this in terms of mentioning the absence of human resources.

Caring for and about patients was expressed as a motivating factor to go beyond scope. This included showing empathy, respect and support for the patient. This is consistent with the findings by Spies et al. (2016), who also found nurses to be able and willing to deliver care to patients in need, by taking responsibility for tasks beyond their usual scope of practice, as long as they had the support to do so. Being compelled to practice outside the scope of practice and simultaneously feeling obliged to help the patient, represents ambiguity in the nurses’ role. It may cause nurses to be in a perpetual emotional quandary, which again will cause stress and justify the need for support.

The difference between practice in a hospital or clinic also became apparent in the study. Participants indicated that nurses were more exposed to working beyond their scope in clinics in rural areas than elsewhere. The current system, whereby nurses can be assigned to any setting and should be able to function in any type of facility, may be reflective of the term ‘general’ nurse, but does not necessarily stimulate the motivation of nurses allocated to settings or facilities where they prefer not to work. This further causes ambiguity in nurses and may lead to demotivation. Similar findings are described by Spies et al. (2016), who found that in Uganda, this was a significant issue in rural areas. Moreover, Miles et al. (2006) state that nursing practice at clinics in Botswana is more autonomous than in hospitals, and present the areas where, for example, nurses prescribing, are more common. Iwu and Holzemer (2017) also found disparities in job satisfaction in relation to the expanded scope of practice across facility levels and types in Nigeria.

Nurses perceived that guidance in terms of boundaries was insufficient. This was related to their job descriptions, lack of resources, parameters of work not being clearly spelt out and the overlap with what they perceived as the role of other health professionals. This caused challenges and created practical dilemmas. A need for protective policies and a clearly defined scope of practice was found in East Africa (Spies 2016; Spies et al. 2016). Job descriptions were often mentioned as the major determinant of one’s scope of practice. Nurses felt affronted by the last part of the job description referred to as ‘any other duties’, which they perceived exposed them to exploitation by the employer. This potential exploitation of nurses is similarly described in literature. Schluter et al. (2011), for instance, found unclear professional responsibilities and blurring of role boundaries between medical officers and RNs, whereby nurses were perceived to be picking up the slack. Zachariah et al. (2009) concurred and pointed to the potential for exploiting vulnerable workers, who might be paid for work for which they are qualified, but they have taken on additional tasks in the context of task shifting for which they are not being compensated, despite a possible increase in workload.

Participants felt they were exposed to dilemmas, such as whether they were legally covered or not, or having to take on additional duties for which they were not necessarily trained. Research indicates that nurses may refrain from expanded practice because of possible legal consequences, time restrictions or lack of remuneration (Fealy et al. 2015). Boundaries, according to Birks et al. (2016), are easier to establish when legislation provides clarity of roles. Likewise, nurses should be cognisant about their boundaries to avoid challenges in distinguishing between what is within and outside their scope of practice. A potential incongruence between legislation and practice may cause inter- and intra-professional conflict, uncertainty, frustration, stress, obstruction to practice (Eagar et al. 2010), and even create a sense of exploitation (Mijovic 2016), which may affect the quality of care being provided and jeopardise health outcomes. A clear description of boundaries, guidelines and protective policies would improve the current situation in which nurses find themselves.

## Strengths and limitations

The sample size of the study has generated sufficient data to contribute to the knowledge, practice and regulatory requirements of nurses in Botswana regarding their scope of practice. Findings have augmented the quantitative component of the study whilst contributing to the development of the scope of practice guidelines. However, the emotive state of nurses, which emerged during the interviews, may have influenced the nature of the participants’ responses. This potential bias was negated by the results of the quantitative component of the study, which corroborated the qualitative findings. Furthermore, participants may have been inclined to overly unload their emotions or frustrations using the researcher as a sound board.

## Recommendations

Nurses should be conversant with their scope of practice. Inadequate knowledge regarding one’s scope may compromise patient care and the legal position of the nurse. To enable informed decision-making by nurses, information sharing is essential (WHO 2020); educators should include and emphasise scope of practice in the curriculum whilst the employer should incorporate comprehensive scope of practice information in the CPD of practising RNs.

Nurses’ attitudes may permeate and affect knowledge acquisition and nursing practice. The employer should consider the cognitive and emotional needs of nurses to enhance and facilitate optimal nursing practice.

Adaptation to practice may cause discomfort or stress. The employer should consider sufficient time and allow an opportunity for nurses to adjust to practice, whilst adequate resources should be put in place for effective and efficient practice. Nurse educators should increase the congruence between education and practice. Compensation and recognition of nurses for expanded and extended practice should also be considered.

Insufficient guidance in terms of scope of practice may lead to frustration, stress and demotivation. The WHO (2020) recommends strengthening the evidence regarding regulatory and governance approaches to enable nurses to practise to their full scope. Nurses need to be provided with clear boundaries in which to practice. The employer and regulatory body should create sufficient and acceptable guidelines and an algorithm to provide nurses with adequate control over their practice.

Nurses provide the bulk of health services in Botswana. In order to continue meeting the health needs of the country, further research should be conducted to identify the degree to which nurses would be willing and prepared to expand their scope of practice, if required, and the conditions and prerequisites for doing so, prior to expanding the scope of practice.

## Conclusion

The qualitative findings of the study indicated a limited understanding of RNs’ knowledge, attitude and practice regarding their scope of practice in Botswana. There was a particularly constrained understanding of the boundaries of their practice and participants further highlighted a need for better guidance regarding these boundaries. Their attitudes appeared to be influenced by varying emotions related to their adaptation and preparation for the expectations in their current roles. It was determined that RNs often practised beyond their scope of practice, related to their knowledge deficits, limited resources and their duty towards their patients. Demands related to practising within or beyond scope were further related to the context in which practice occurred, with more demands in clinics with limited resources. The limited knowledge and need to adapt their scopes of practice because of contextual demands leave nurses feeling vulnerable and exposed. They expressed a need for improved guidance regarding their scope of practice. In order to increase congruence between actual and regulated practice, guidelines are warranted to facilitate the provision of adequate knowledge, improve attitudes and ensure safe practice within established boundaries of practice.
